# MAID : An effect size based model for microarray data integration across laboratories and platforms

**DOI:** 10.1186/1471-2105-9-305

**Published:** 2008-07-10

**Authors:** Ivan Borozan, Limin Chen, Bryan Paeper, Jenny E Heathcote, Aled M Edwards, Michael Katze, Zhaolei Zhang, Ian D McGilvray

**Affiliations:** 1 Banting and Best Department of Medical Research, University of Toronto, 112 College St, Toronto, ON M5G1L6, Canada; 2 Department of Medical Genetics and Microbiology, University of Toronto, 1 King's College Circle, Toronto, ON M5S1A8, Canada; 3 Department of Medical Biophysics, University of Toronto, 610 University Avenue, Toronto, ON M5G2M9, Canada; 4 Toronto General Research Institute, 610 University Avenue, Toronto, ON M5G2M9, Canada; 5 Department of Surgery, Faculty of Medicine, University of Toronto, 1 King's College Circle, Toronto, ON M5S1A8, Canada; 6 Toronto Western Hospital, 399 Bathurst St, Toronto, ON M5T2S8, Canada; 7 Department of Microbiology, Box 357242, University of Washington, Seattle, WA 98195-7242, USA

## Abstract

**Background:**

Gene expression profiling has the potential to unravel molecular mechanisms behind gene regulation and identify gene targets for therapeutic interventions. As microarray technology matures, the number of microarray studies has increased, resulting in many different datasets available for any given disease. The increase in sensitivity and reliability of measurements of gene expression changes can be improved through a systematic integration of different microarray datasets that address the same or similar biological questions.

**Results:**

Traditional effect size models can not be used to integrate array data that directly compare treatment to control samples expressed as log ratios of gene expressions. Here we extend the traditional effect size model to integrate as many array datasets as possible. The extended effect size model (MAID) can integrate any array datatype generated with either single or two channel arrays using either direct or indirect designs across different laboratories and platforms. The model uses two standardized indices, the standard effect size score for experiments with two groups of data, and a new standardized index that measures the difference in gene expression between treatment and control groups for one sample data with replicate arrays. The statistical significance of treatment effect across studies for each gene is determined by appropriate permutation methods depending on the type of data integrated. We apply our method to three different expression datasets from two different laboratories generated using three different array platforms and two different experimental designs. Our results indicate that the proposed integration model produces an increase in statistical power for identifying differentially expressed genes when integrating data across experiments and when compared to other integration models. We also show that genes found to be significant using our data integration method are of direct biological relevance to the three experiments integrated.

**Conclusion:**

High-throughput genomics data provide a rich and complex source of information that could play a key role in deciphering intricate molecular networks behind disease. Here we propose an extension of the traditional effect size model to allow the integration of as many array experiments as possible with the aim of increasing the statistical power for identifying differentially expressed genes.

## Background

Microarray technology is becoming an important tool for biological research and clinical diagnostics [1], but it has the reputation of being noisy: studies addressing the reproducibility and reliability of microarray data across different laboratories and platforms have resulted in inconsistent results. Some have found agreement between experiments [2-7] while others have not [8-11]. A study by Irizarry et al. [12] on microarray data reproducibility has demonstrated that disagreement observed in some of the studies may be also due to questionable statistical analysis. There is general agreement that the variability inherent to DNA microarray technology is due to the following factors. There are a number of microarray platforms independently developed by industry and academia. Different protocols are used by different laboratories for RNA preparation and labeling. Different statistical and computational tools are used in the analysis of the microarray results. Due to these differences it is challenging to extract reproducible, biologically meaningful information from different DNA microarray experiments that address the same, or very similar biological questions. One possible solution to extract this information is to use meta-analysis methods that integrate the results of separate studies in a statistically meaningful manner. There are two main types of meta-analysis that are commonly used for microarray data integration. The first consists of integrating summary measures of gene expression measurements across studies. The advantage of this type of approach is that it avoids the need for estimating the inter-study variability and the issue of cross-platform normalization. Rhodes et al. [13] were the first to implement this type of approach. This group implemented a statistical model based on integrating p-values from individual studies to estimate the overall p-value for each gene across studies. The authors integrated four published prostate cancer gene array studies. Many of the genes identified were confirmed to be components of biologically relevant pathways, implying that the method extracted biologically useful information. Subsequently Parmigiani et al. [14] proposed a different model that uses a correlation-based method to search for consistent gene expression patterns across multiple studies. They demonstrated that their method can improve correlation of gene expressions across studies. Rather than combining p-values or correlations the second type of meta-analysis consists of integrating gene expression measures across studies. Choi et al. [15] were the first to propose this type of approach using an effect size measure [16] with a method that explicitly models the inter-study variability. Using the same datasets as those used in [13], they demonstrated that their method led to increased sensitivity and reliability. Subsequently Hu et al. [17] extended this model by incorporating a quality measure for each gene in each study into the effect size estimates. Using their model the authors combined two lung cancer Affymetrix datasets generated from two different laboratories and found that their method identifies more differentially expressed genes than previous methods. Taken together these studies suggest that a subset of biologically plausible and statistically significant genes can be determined from the integration of different array technologies. With an ever-increasing amount of microarray data being produced it is critical to develop statistically sound methods that will efficiently integrate, evaluate and cross-validate as many array experiments addressing the same biological question as possible. Even though progress has been made in integrating various array datasets, challenges still remain, one of which is that all the existing methods require experiments with two separate groups of data.

A two channel microarray technology continues to be used as one of the most common platforms for gene expression profiling [18,19]. One experimental approach using two channel arrays is to directly compare levels of mRNA expression between treatment and control samples (also known as direct experimental design). Such experiments lead to datasets with only one group of gene expression ratios. The method proposed in [15] can not be applied to such datasets since it requires two groups of data. In order to allow the integration of as many datasets as possible, including experiments with one group of data, we extend the model proposed in [15] and propose a new mathematical framework for integrating microarray experiments with one group, two groups of data or mixed groups.

The model proposed in our study is more general than the model proposed in [15], and allows the integration of microarray data of any type generated across different laboratories, platforms and experimental designs. As such, it provides more flexibility for microarray data integration than the previously published effect size based model. The model provides also a new mathematical framework for addressing the inter-study variation for microarray data of different types.

## Results

In order to assess the usefulness of our model to integrate real data we applied our method to three different expression datasets generated from two different laboratories using three different 2-channel array platforms and two different experimental designs. All three datasets compared normal liver tissue to liver tissue chronically infected with hepatitis C virus (HCV).

### Exploratory data analysis

**Figure 1:**
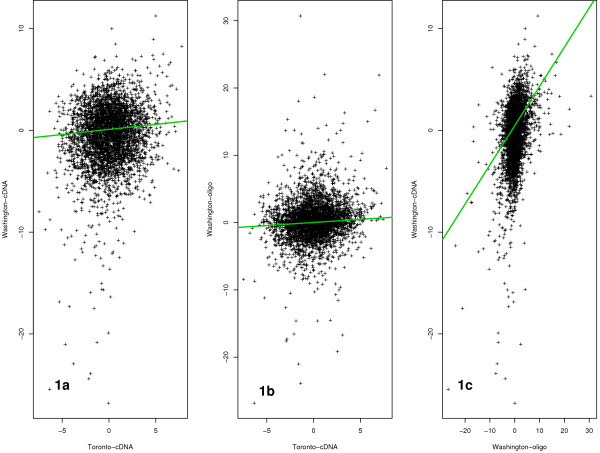
**Effect size correlations between experiments**. Correlation plots of effect sizes between three experiments; 1a) R = 0.13 (Toronto-cDNA vs Washington-cDNA), 1b) R = 0.14 (Toronto-cDNA vs Washington-oligo), and 1c) R = 0.38 (Washington-oligo vs Washington-cDNA).

**Figure 2:**
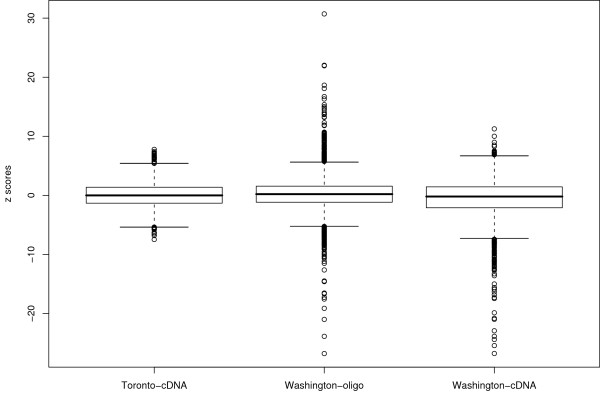
**Boxplot of effect sizes for the three experiments**. Boxplot showing z scores of the three experiments to be centered around zero indicating that the three experiments are measuring similar differences between conditions.

Before data integration was carried out, an exploratory data analysis as proposed in [20] was conducted to determine if there were any fundamental differences between experiments that would preclude data integration. As shown in Figure 1, low correlation coefficients were observed between estimated effect sizes of the three studies: $R_{\left(T_{cDNA}\;vs\;W_{cDNA}\right)}=0.13$, $R_{\left(T_{cDNA}\;vs\;W_{oligo}\right)}=0.14$ and $R_{\left(W_{oligo}\;vs\;W_{cDNA}\right)}=0.38$. These low correlation coefficients highlight differences between the three experiments. In high-throughput microarray experiments, a common expectation is that the majority of genes in each study will show little or no difference between conditions. Figure 2 shows the distributions of z scores (see Methods section eq.15) in the three experiments, all of which are centered around zero. This finding indicates that most of the genes in each experiment show little or no differences between treatment and control samples. A significant deviation from zero in any of the three datasets, due to some large systematic effect, would be indicative of fundamental differences between experiments that could not be solved by statistical means. Thus even when low correlations between experiments are observed (for example due to a large number of genes having log2 expressions close to zero with random measurement error) this does not automatically imply that small sets of genes with significant effects across experiments would not be observed and that data integration should not be considered.

**Figure 3:**
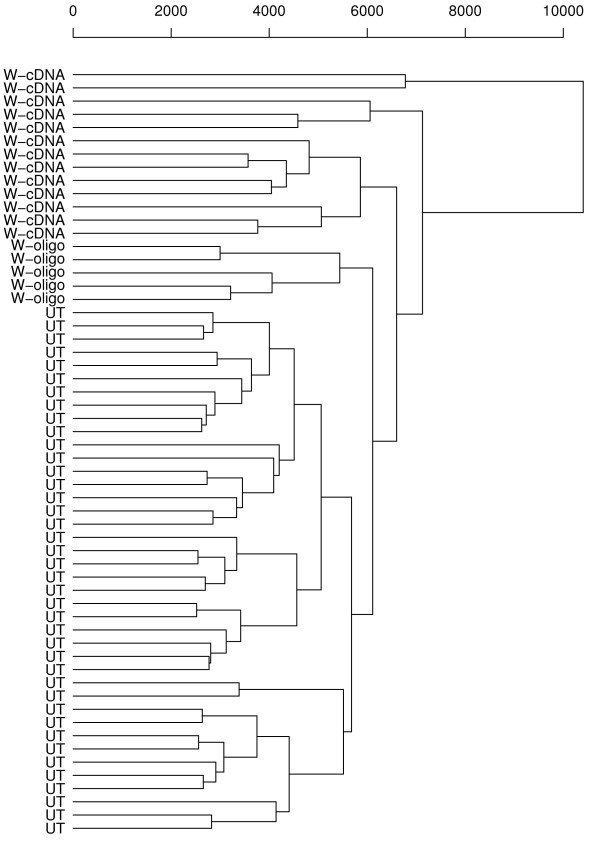
**Unsupervised hierarchical clustering analysis**. An unsupervised hierarchical clustering of individual samples was performed using relative gene expression (i.e ratios of gene expressions in HCV and normal tissue). Samples cluster according to each individual platform, indicating the presence of intra-study variability due to lab/platform effects (UT designates cDNA arrays/samples from University of Toronto, W-cDNA designates cDNA arrays/samples from University of Washington, and W-oligo designates oligo arrays/samples from University of Washington).

By adopting a similar approach to Kim et al. [36] we present a cluster plot in Figure 3 that shows a relationship between the three datasets before data integration. We find that if clustering of individual samples is done using relative gene expressions (i.e expressions of genes in HCV to normal tissue), the samples cluster according to each individual platform, indicating the presence of intra-study variability due to lab/platform effects.

**Figure 4:**
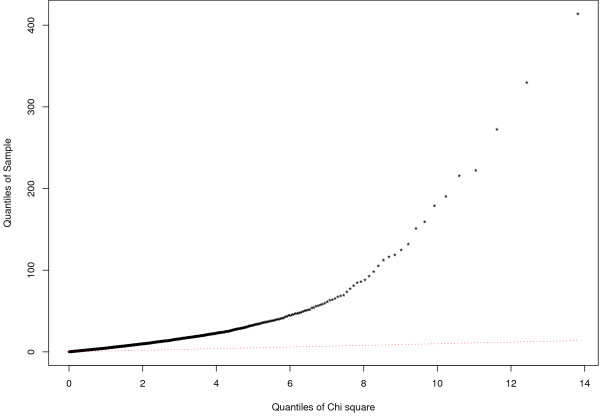
**Quantile-Quantile plot**. Gene by gene testing for the homogeneity of study effects. Overall test results are shown by the Quantile-Quantile plot of the observed (black curve) vs expected Q quantiles (red curve), the expected Q values are from the $\chi_{\left(l-1\right)}^{2}$ distribution, where l designates the number of experiments. The difference between the observed and the expected Q quantiles are large and show that a random effect model should be considered for data integration.

**Figure 5:**
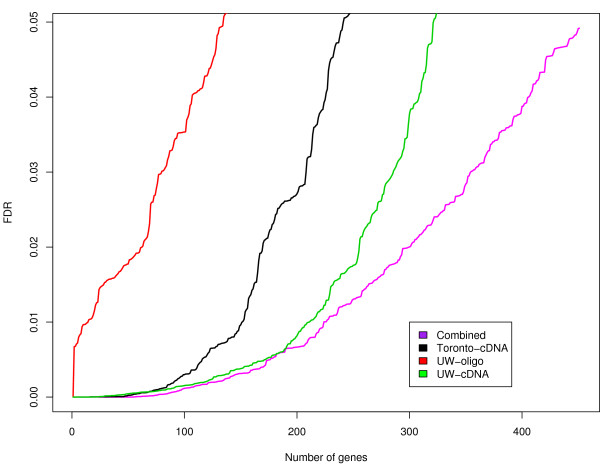
**Meta-analysis false discovery rate**. The number of genes vs their significance for individual studies and for the integrated study.

In order to test for homogeneity between datasets, we used the Cochran Q statistics given in eq.10 (see the Methods section). The results of the test are shown in Figure 4. The observed Q values from the three experiments deviate significantly from the expected quantiles of the $\chi_{\left(l-1\right)}^{2}$ distribution, suggesting that the three datasets are heterogeneous. Heterogeneity indicates significant variability between studies that requires a random effect model (REM) to be fitted. When applied to our pre-processed datasets, the REM model found a set of 451 significant genes with FDR ≤ 0.05. In order to asses the advantage of integrating these three datasets together, we first determined the number of genes that had an FDR ≤ 0.05 in the meta-analysis study but for which the FDR in all three studies was higher than the FDR in the meta-analysis study. Of the total of 451 genes in the meta-analysis study, we found 237 to satisfy this criterion. We designated these genes as integration-driven discovery (IDD) genes as first introduced in [15]. Figure 5 shows a plot of the gene number versus FDR (FDR ≤ 0.05) for each independent dataset and demonstrates that the largest number of significant genes is observed in the meta-analysis. This increase in the number of significant genes is an indication of the potential benefit in integrating these three datasets using our model.

### Genes determined to be significant in the meta-analysis model

In order to further assess the advantage of data integration, we decided to examine whether genes found in our analysis had direct biological relevance. Genes that are determined to have statistically significant expression level changes may still have low fold increases (or decreases) that might not be biologically relevant. Although there is no consensus in fold increase/decrease associated with 'biological relevance', we chose a fold change of at least 1.5 (increased or decreased) between HCV and normal in at least one of the three integrated studies based on the estimate of the median standard deviation (median sd = +/- 0.23) of fold gene expression measurements in the three experiments (a 1.5 fold cuto3 on gene expression levels is 2 standard deviations away from the mean of genes with no expression (i.e fold = 1), and thus is less likely to be confounded with non-expressed genes). We found a total of 206 genes to satisfy those criteria. Of the 206 genes, 79 genes were integration-driven discovery (IDD) genes as defined in [15]. We have used a 1.5 fold cutoff in our previous array studies using clinical samples and have determined a number of biologically-relevant effects (Chen et al. [31], Borozan et al. [30], Chen et al. [37]), and were able to validate 85 % of genes expressed at the 1.5 fold level, using quantitative real time – PCR (for more detail about gene validation we refer the reader to [30,31]).

### Biological pathway analysis

In order to determine the biological themes overrepresented in our gene list, we used the R package GOstats [21]. GOstats searches for overrepresented GO biological themes by determining if a given GO category contains more genes determined to be significant in a given experimental condition than one might expect by chance. Taking p-values *<*0.05 for significant overrepresentation we found a number of enriched GO biological processes to be associated with HCV infection including: immune response, defense response and response to virus. Many of the genes in each of the enriched GO categories (see Table 1 for the top ten GO categories (see Additional file 1, for the full table of the GO over-represented BP categories)) have been found to play key roles in host antiviral response to HCV infection [22], a number of which are interferon stimulated genes (data not shown here).

**Table 1:** Significantly over-represented GO biological processes

**GO over-represented categories**	**GOBPID**	**P value**	**OddsRatio**	**ExpCount**	**Count**	**Size**
immune response	GO:0006955	8.39E-008	3.56	11.25	31	196
organismal physiological process	GO:0050874	1.35E-007	2.69	24.44	50	426
response to biotic stimulus	GO:0009607	4.71E-007	3.18	12.74	32	222
defense response	GO:0006952	4.76E-007	3.24	12.11	31	211
response to stimulus	GO:0050896	2.75E-005	2.15	28.23	49	492
regulation of caspase activity	GO:0043281	3.20E-004	22.41	0.4	4	7
response to pest, pathogen or parasite	GO:0009613	1.01E-003	2.72	6.83	16	119
response to other organism	GO:0051707	1.32E-003	2.64	7	16	122
physiological process	GO:0007582	4.77E-003	2.2	146.25	157	2549

Shows the top ten over-represented GO biological process categories obtained from the list of genes determined to be significant using MAID (Micro Array Data Integration Model). The over-represented GO categories are ordered according their statistical significance in decreasing order (Count designates for each GO category term tested, the number of genes from the significant gene list that are associated with that term, Size designates the total number of genes associated with each GO term tested and ExpCount designates the expected number of genes obtained by chance alone for each GO term tested).

In order to determine if particular biochemical pathways were enriched in genes from our list of 206 genes, we performed a KEGG pathway database [23] query using the R package GOstats [21]. We identified five significantly enriched KEGG pathways termed; "Antigen processing and presentation" (p-value ≤ 8.2e-05), "Type I diabetes mellitus" (p-value ≤ 1.3e-03), "Ribosome" (p-value ≤ 3.8e-03), "Toll-like receptor signaling pathway" (p-value ≤ 4.4e-02) and "Linoleic acid metabolism" (p-value ≤ 4.7e-02) using the Hypergeometric test of GOstats package (see Table 2). Three of the five enriched pathways found in our analysis have been directly associated in previous studies with HCV. Genes from KEGG's "Antigen processing and presentation" pathway were associated with HCV persistence in infected individuals [24], genes from the "Ribosomal" pathway were shown to interact with the virus RNA internal ribosomal entry site [25-27], while genes from "Toll-like receptor signaling pathway" have been shown to be modulated by HCV proteins in liver cells [22]. These findings indicate that our model is selecting genes that are enriching pathways relevant to HCV infection. More importantly two of the five enriched KEGG pathways ("Antigen processing and presentation" and "Type I diabetes mellitus") obtained through data integration were found not to be enriched in any of the three individual studies, in other words these pathways were identified purely by means of meta-analysis. They are an example of how weak but consistent signals across the three studies are brought together in order to achieve more reliable results, highlighting the effectiveness of our integrative approach. Because many of the genes found in this study are of direct relevance to the Hepatitis C disease, a more detailed study of biological implications of our findings will appear in a separate paper.

**Table 2:** Significantly over-represented KEGG pathways

**KEGG over-represented pathways**	**P value**
Antigen processing and presentation	8.16E-005
Type I diabetes mellitus	1.26E-003
Ribosome	3.76E-003
Toll-like receptor signaling pathway	4.40E-002
Linoleic acid metabolism	4.65E-002

Shows over-represented KEGG pathways obtained from the list of genes determined to be significant using MAID (Micro Array Data Integration Model).

### Comparison of MAID with other integration methods

In this section we compare results from data integrated with MAID to results integrated with the other methods mentioned in the Background section. Among four proposed methods for microarray data integration [13-15,17], only two methods based on combining summary measures (Rhodes et al. [13] and Parmigiani et al. [14]) can be applied to datasets with both one and two groups of data (a single group of data with two-color array technology can be produced using a direct design approach where disease and control samples are co-hybridized on the same array). The second index introduced in our model allows the general framework proposed in [15] to be extended and applied to datatypes that previously could not be integrated with this method.

**Figure 6:**
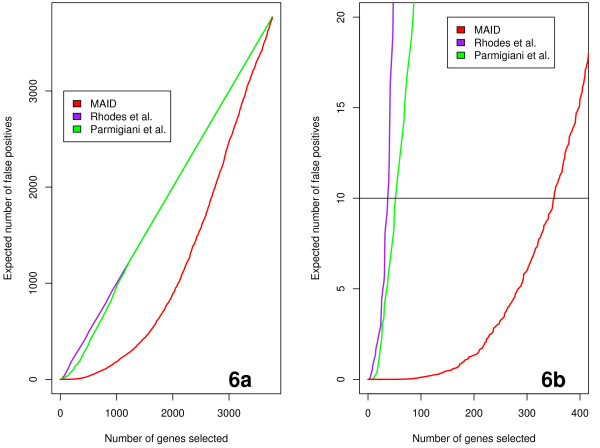
**False discovery rate for the three integration methods**. Figure 6a shows the plot of the number of genes selected by each method versus the expected number of false positives (*E*(*F P*)), figure 6b shows the same plot as figure 6a with the expected number of false positives *E*(*F P*) ≤ 21.

In order to compare results obtained from these three different models we compared gene lists selected as significant by each individual method. In order to make a valid comparison the selected gene sets were required to have the same expected number of false positives *E*(*F P*), in this way the comparison between results obtained with MAID and results obtained with other models is ensured to be done at the same statistical significance level (see Tables 3, 4 and Figure 6). For the purpose of comparison we chose a reasonably conservative value for *E*(*F P*) of 10 (see also Figure 6b). The biological relevance of gene sets selected by each individual model is then evaluated by comparing the significance, the biological relevance and the content (i.e gene number) of enriched GO biological process categories.

**Table 3:** The number of significant genes selected by each of the three integartion models with *E*(*F P*) = 10

	**# of genes**	**# of genes**	**# of genes**
	**(Rhodes et al.)**	**(Parmigiani et al.)**	**(MAID)**
**E(FP) = 10**	37	50	350
**E(FP) = 10 and |fold| ≥ 1.5**	37	14	159

The number of significant genes selected by each model. The first row designates the number of genes selected by each model with the expected rate of false positives *E*(*F P*) = 10. The second row designates the number of genes from the first row that are either up (or down) regulated in HCV samples compared to Normal with |fold| ≥ 1.5

**Table 4:** Top five over-represented GO biological process categories obtained with three different integartion models

**GO (BP) categories**	**P values**	**# of genes**
**MAID**		
organismal physiological process	8.90E-007	41
immune response	4.96E-006	24
response to biotic stimulus	1.41E-005	25
defense response	1.81E-005	24
response to stimulus	1.98E-004	39
**Rhodes et al.**		
response to biotic stimulus	8.12E-006	11
immune response	1.84E-005	10
response to pest, pathogen or parasite	2.12E-005	8
response to other organism	2.54E-005	8
defense response	3.54E-005	10
**Parmigiani et al.**		
glyoxylate metabolism	4.44E-003	1
sphingolipid catabolism	4.44E-003	1
sphingomyelin metabolism	4.44E-003	1
sphingomyelin catabolism	4.44E-003	1
coagulation	8.37E-003	2

Top five enriched GO categories obtained with, MAID, Rhodes et al., and, Parmigiani et al. In each case genes had to be up (or down) regulated |fold| ≥ 1.5 in HCV samples compared to Normal with an expected number of false positives *E*(*F P*) = 10 for each gene list.

In Figure 6 we show the plot of the number of genes selected by each individual model versus the expected number of false positives. We found that the MAID model selects more genes when compared to the other two models for the same expected rate of false positives (i.e *E*(*F P*) = 10, see also Figure 6b). For the purpose of clarity Figure 6b shows the same plot as Figure 6a but limited to gene lists with the expected number of false positives *E*(*F P*) ≤ 21. The number of significant genes selected by each model is given in Table 3.

In order to asses whether the larger gene set selected by the MAID model (for the same expected false positive rate *E*(*F P*) = 10) enriches relevant biological categories we compared the enriched GO biological process (BP) categories obtained from gene lists selected by each individual model. We also imposed a threshold on selected genes' fold changes by requiring genes to be up (or down) regulated by |fold| ≥ 1.5 (for the reasons noted earlier) in HCV samples when compared to Normals (see Table 3). In Table 4 we give the top 5 enriched GO categories from each model.

As shown in Table 4 enriched GO (BP) categories obtained with a correlation-based method proposed by Parmigiani et al. [14] are less significant than categories obtained from either MAID or the model proposed by Rhodes et al. [13], and contain many fewer genes (no more than two per category) that show no clear relevance to the HCV disease.

Of the top five significantly enriched GO (BP) categories obtained with gene sets selected by the model proposed by Rhodes et al. [13] and MAID, two can clearly be associated with HCV disease; these are "immune response" and "defense response" (see Table 4). Table 4 shows that the enrichment in genes selected by the MAID model is higher for both of these categories "immune response" p-value = 4.96e-6 (MAID) vs p-value = 1.84e-5 (model from Rhodes et al. [13]) and "defense response" p-value = 1.81e-5 (MAID) vs p-value = 3.54e-5 (model from Rhodes et al. [13]). These results indicate that when gene sets selected by the model from Rhodes et al. [13] are compared by those selected by MAID, the larger MAID gene set improves the enrichment significance of the two of the most significant and HCV relevant GO categories and points to an increase in statistical power when compared to the model proposed by Rhodes et al. [13].

**Figure 7:**
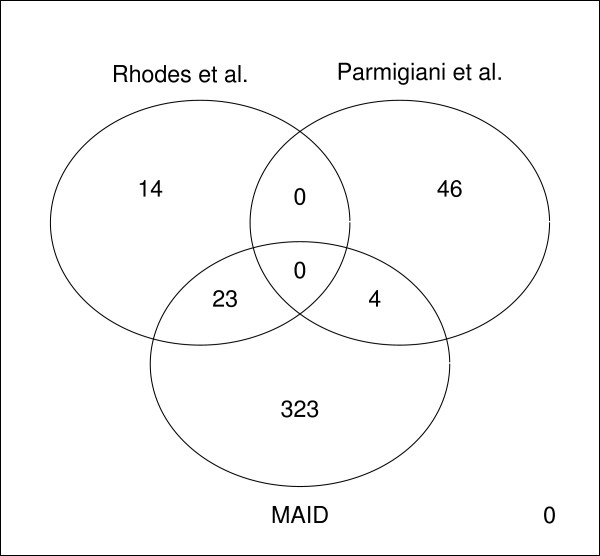
**Overlap of genes found to be significant by each of the three integration methods**. Overlap among top genes selected from each of the three methods with *E*(*F P*) = 10.

As shown in Figure 7 the highest overlap in genes selected by each individual model was observed between the MAID model and the model proposed by Rhodes et al. [13] (23 genes) while the lowest overlap and highest discrepancy in genes and GO enriched categories was observed when comparing results obtained with either the MAID model or the model of Rhodes et al. [13] with the correlation-based method proposed by Parmigiani et al. [14]. The overlap in genes between the model proposed by Rhodes et al. [13] and the model proposed by Parmigiani et al. [14] and the overlap between MAID and the model proposed by Parmigiani et al. [14] are both very low (zero and four genes respectively).

## Discussion

In this study we introduce a new effect size based model for microarray data integration. We demonstrate that our model, together with appropriate data pre-processing methods, can be used to integrate expression data across different laboratories, array platforms and experimental designs that results in an increase in statistical power for identifying differentially expressed genes when integrating data across experiments. Moreover, we show that genes selected as significant by our model enrich relevant biological pathways and processes.

In order to obtain the best possible results with our model, a number of important problems relating to each individual data set had to be addressed. First, it is only reasonable to integrate experiments that aim to address the same or similar biological questions. In order to address the problem of matching of samples and experiments, we integrated only experiments that compared samples of same biological type. Second, because most of the disagreement between individual array experiments was found to be due to platform-dependent probe effects [12], we decided to use only relative gene expression ratios instead of absolute measurements. Third, in order to ensure better agreement between gene annotations across platforms, we focused only on genes that had identical annotation entries in the NCBI Entrez Gene database.

After addressing the problem of matching of probes, samples and experimental conditions we used exploratory analysis methods proposed in [20] to determine if data from the three experiments presented any important systematic bias that would preclude their integration. We found all three datasets to show low correlation coefficients between their effect sizes – though a slightly higher correlation coefficient was found for datasets from the Washington group (see Figure 1). However, inspection of individual effect size distributions showed no fundamental differences between the three datasets (see Figure 2). Low correlations of effect sizes could result from a small group of genes showing similar effects across the three experiments. When expression measurements were integrated using the above methodology, we found 451 genes to be significantly expressed across all three studies with a false discovery rate (FDR) ≤ 0.05. Of these 237 had higher statistical significance in the integrated study than in any individual study. Of 79 integration driven discovery genes found with absolute fold expression greater than 1.5, 57 were shown to be up-regulated (or down-regulated) by at least 1.5 fold in only one of the three studies. This result suggests that the magnitude of fold increase (or decrease) in each individual experiment is a poor indicator of the overall gene activity when comparing across experiments and that a more suitable metric such as effect size needs to be used. Furthermore, of the 206 genes that were found to be significant (with fold ≥ 1.5) in our analysis, 11 were found not to be significant in any of the individual studies. The potential involvement in HCV disease of these genes identified through meta-analysis alone will require further biological study. Of four previously published methods proposed for microarray data integration [13-15,17], two methods [13,14], based on combining summary measures, can be applied to datasets generated with mixed groups (i.e with two groups and a single group of data). Comparing results obtained with MAID to results obtained with the models proposed by Rhodes et al. [13] and Parmigiani et al. [14], we found that MAID selects more genes than any of the summary statistics based methods, and that additional genes selected by MAID are relevant to the HCV disease. Genes selected by MAID produce an increase in enrichment of relevant HCV GO categories when compared to results obtained with the two summary statistics methods (see Table 4). These findings argue that MAID produces less conservative results that are also biologically more relevant, indicating an increase in statistical power.

The overlap in results of the top genes selected by each method (for exactly the same number of expected false positives) indicates that models based on integrating p-values [13] and effect sizes (i.e MAID), across experiments, give more similar results than the model based on integrating gene correlations [14].

Models based on summary statistics that integrate p values [13] or expression correlations [14] across studies can be used to obtain more precise estimates of significance of gene expressions than those obtained from the individual array studies (see for example [13]). However such approaches do not take into account the inter-study variability and can produce results that are significant even for genes that have significant fold changes but that are observed to be expressed in opposite directions (increased versus decreased) across studies. Models that do take the inter-study variability into account, such as Choi et al. [15] and MAID, would not consider such changes as significant (for example data integration using the model proposed by Rhodes et al. [13] leads to 19 genes that are significant but for which the fold increase/decrease is directed in opposite directions by at least 1.5 fold in at least one of the studies). In addition to ignoring the direction of change in gene expressions across studies, summary-statistics based models do not take the magnitude of observed effects (i.e fold changes) into account either. In this way significant statistical changes (or small p values) might not necessarily correspond to important biological effect (i.e fold changes) and could inflate the number of false positives. Effect size based models instead, integrate data directly by taking into account the magnitude of the effect and its consistency both within and across studies. Moreover it has been shown that models based on integrating summary statics are less sensitive to small but consistent expression changes than an effect size based model (see Choi et al. [15]).

Though we agree in principle with the approach proposed in Choi et al. [15], we note that the model assumes that a fixed or random effect model should be fitted for all the genes. However, this approach might not always be appropriate. As pointed out in [20], it is more likely that for some genes there would be no effect observed, while for others a fixed or random effect model would be more appropriate. A more flexible approach should improve the sensitivity and reliability of this model. Furthermore, as noted in [15], for microarray data and biological systems in general, genes can not always be assumed to act independently, but often show dependency through interactions and correlations. Without a better understanding of gene-gene interaction structures, it is difficult to realize how such improvements could be included in the model. We also note that particular care needs to be taken when integrating many small-sized microarray studies with this model as the estimated between study variability *τ*^2 ^will be biased and would influence overall results [20,28].

The approach proposed in our study differs from that of [15] and the GeneMeta algorithm [21] in several important aspects. The set of methods proposed in [15], as implemented in the GeneMeta [21] algorithm, can only be applied to experiments with two separate groups of data and thus can not be applied to two-channel microarray experiments measuring differences in gene expression values between treatment and control groups using a direct experimental design. In order to integrate as many microarray datasets from the public domain as possible we proposed a new integration method which we implemented in form of the R package MAID (we have made every effort possible to provide an R package with an easy to understand, high-quality documentation for non-expert R users, the package is available upon request from the corresponding authors and will be submitted to the Bioconductor [21] project to ease access and dissemination).

In MAID the type of analysis applied depends on the type of data analyzed. Thus for microarray experiments with two groups of data we use the standard effect size model proposed in [15]. For microarray experiments with one group of data we propose a second standardized index based on the paired *t*-statistic (see eq.6 in Methods section) which follows a Student's *t*-distribution times $\sqrt{\frac{1}{n}}$, with (*n *- 1) degrees of freedom (where n is the number of microarray replicates).

In addition to eq.6 (see Methods section) we also propose new estimators for both the pooled standard deviation (which is now given in eq.7 and which replaces the pooled standard deviation given in eqs.2–3 in the Choi et al. model) and the estimated variance (which is now given in eq.8 and which replaces the estimated variance for the unbiased effect size given in eq.5 in the Choi et al. [15] model).

Although we adapt the same general hierarchical model framework as described in Choi et al. [15], a major difference is that for direct design experiments the inter-study variability given in eq.12 (first proposed by DerSimonian and Laird [29]) is calculated using new expressions for the pooled standard deviation and the estimated variance given in eq.7 and eq.8, instead of the expression given by Choi et al. in eq.3 and eq.5 (see Methods section).

The same changes occur in eqs.9–15 with new estimators replacing those described in Choi et al [15]. Depending on the type of datasets integrated the homogeneity test is calculated using either one or both types of standardized indices and their respective variances. MAID implements a permutation method that is specific for each data type, experiments with two groups of data are considered as a two class label case, while experiments with one group of data are considered as a one class label case. In addition to the permutation method for a two class label case, MAID implements a second permutation method (a feature which did not exist in the model proposed by Choi et al.) for a single class label case necessary in the calculation of false discovery rate (FDR) (see eq.16 in Methods section). Without the proposed new estimators given in eqs.6–8 (see Methods section) and their implementation through eqs.10–16 (see Methods section) it would not have been possible to integrate array experiments with both direct and indirect designs using a more sophisticated model, such as the one proposed in this study that takes both the intra and inter-study variability into account.

## Conclusion

Traditional effect size models [15] are limited to integration of array datasets with two groups of data. Here we extend the traditional effect size model in order to increase the sample size by allowing the integration of array experiments of any type. Using our model we have shown that it is possible to detect small but consistent changes in gene expression across these three biologically similar but independent studies. Genes with weak signals in each individual experiment can be seen as potential false negatives. We have shown that the number of false negatives can be decreased effectively by using our model. We have also demonstrated that a sizable number of genes could be cross-validated through inter-study comparison indicating that these studies show a certain degree of reproducibility. Our results also indicate that technical and biological variability present in datasets obtained from different laboratories, different platforms and designs can be overcome by appropriate data pre-processing and meta-analysis methods. By comparing our model to other integration methods available, we show that our model selects more genes (for the same number of expected false positives) that are of direct biological relevance to the experiments under consideration.

Finally we have shown that most of the genes found to improve in significance after data integration with our model are of direct biological relevance to the three experiments. High-throughput proteomics and genomics data provide a rich and complex source of information which may help to decipher the complex molecular networks behind disease. Beyond the analysis of the gene expression data presented in this study, our model provides a way of integrating multiple microarray datasets across a broad range of cross-platform studies, and allows a more general and flexible framework for microarray data integration.

## Methods

### Data sources and preprocessing

Three microarray expression datasets from two laboratories were collected. Two datasets were obtained from the University of Washington. These datasets were collected using two different versions of Agilent array technology. One dataset was generated using two-channel Agilent Human 1 cDNA array platform containing 12,814 probes. This study used a direct design and included 13 chronic HCV samples co-hybridized with 13 normal samples. The second dataset was generated using two-channel Agilent Human 1A (V2) 60-mer oligonucleotide array platform containing 20,173 probes. This study used direct design and included 5 chronic HCV samples co-hybridized with 5 normal samples. The third dataset was obtained from the University of Toronto UHN Microarray Liver Disease Project [30,31]. This dataset was generated using two-color UHN cDNA microarray slides containing 19000 probes. This study used indirect design and included 40 chronic HCV samples co-hybridized to reference RNA and 20 normal samples co-hybridized to the same reference RNA. In total 78 samples were collected across the three studies. All arrays from University of Washington group were normalized using the Rosetta Resolver error model [32] while all arrays from University of Toronto were normalized using an R-based, intensity-dependent LOWESS scatter plot smoother (see the Methods section of [31]). Prior to data-integration all expression data were log2 transformed.

#### Annotation

**Figure 8:**
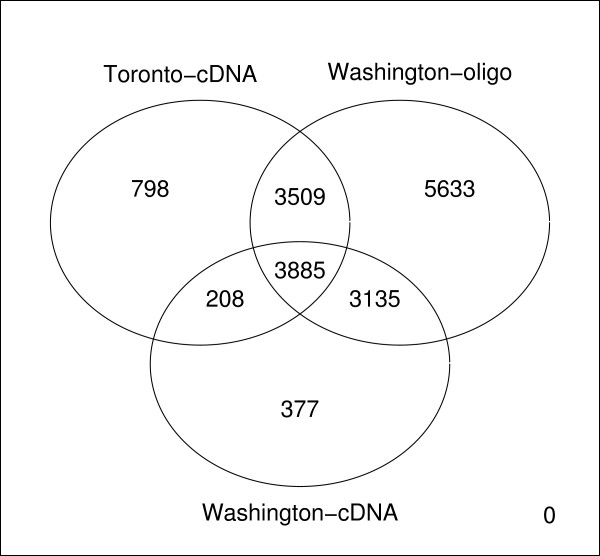
**Gene annotation overlap between experiments**. Venn diagram showing agreement between annotations across three different platforms using Entrez Gene identifiers.

In order to assure the best possible match for features across different microarray platforms, we used mappings that matched each feature to genomic information available from the NCBI Entrez gene database. We used the R package AnnBuilder [21] to match probe GeneBank identifiers provided by each manufacturer to Entrez Gene identifiers from NCBI. Only probes that had an entry for each platform were considered for further analysis. Figure 8 is a Venn diagram demonstrating the degree of agreement between each platform using Entrez Gene identifiers. Only probes that have been annotated and for which the measured intensity was available after image and data processing in each of the three studies were considered for further analysis. A list of 3885 genes was found to be consistently annotated between the three studies (see Figure 8); of those 3770, were found to have intensity measurements across all studies. The gene annotation overlap found in our study is similar to the gene annotation overlap found in a cross-study comparison of three different experiments done on different platforms (two channel cDNA arrays and single channel Affymetrix arrays) [14].

#### Data integration with models proposed by Parmigiani et al. and Rhodes et al

We used the R package MergeMaid [21] to integrate the three dataset using the integration model proposed by Parmigiani et al. [14].

The integration model proposed by Rhodes et al. [13] was implemented in R as follows. Gene specific p-values were computed separately for each study and combined using the Fisher summary statistics *S *as shown in equation below

$$S=-2\sum_{j=1}^{l}log(p_{j}),$$

where *p*_*j *_is the gene specific p-value for the *j*^*th *^study. The summary *S *statistics is then compared to an empirical distribution, obtained by computing summary statistics *S *from 100000 random permutations of p-values from each study. The meta-analysis p-value are computed as the proportion of random *S *statistics larger than the actual *S *statistics. To estimate the false discovery rate we used the R package qvalue [21,33] with the *λ *parameter set to zero that produces an estimate of FDR according to the methodology proposed by Benjamini and Hochberg [34].

### Microarray Data Integration Model (MAID)

#### Effect size

Microarray experiments are done using either two-channel (e.g. custom or commercially available cDNA or oligonucleotide arrays) or single channel arrays (e.g Affymetrix). For two-channel arrays the experimenter can chose between either direct or indirect designs. There are several distinct steps involved in producing two-color microarrays. In a direct design for two color cDNA microarrays, treatment and control target mRNA samples are (*i*) reverse-transcribed into cDNA (*ii*) labeled with different fluorescent dyes (usually red-fluorescent dye, Cy5 and green-fluorescent dye Cy3), (*iii*) mixed in equal proportions and hybridized to DNA probes on the glass slide. In the case of indirect design a common reference is used on all the arrays. The basis for the model used in our analysis was first proposed by Choi et al. [15]. A recent implementation of this model using the R package GeneMeta [21] allows experiments with two separate groups of data to be integrated. This model cannot be applied to two-channel microarray experiments done using a direct design approach in which only one group of data is available. Limiting data integration to experiments with two separate groups of data would be wasteful of other potentially valuable data sets. In this study we describe a new model implemented in form of the R package MAID (Microarray Data Integration Model) that can integrate two-channel array experiments with both direct and indirect designs (the R package MAID is available from the corresponding authors upon request). For experiments with two groups of data we follow the implementation of the GeneMeta [21] algorithm. For the case where only one group of data is available we introduce a new standardized index as a measure of the mean difference between treatment and control samples. The model in [15] and GeneMeta [21] use a well-known effect size estimator [16] as a standardized measure of mean differences between treatment and control groups. The expression for the effect size is given in eq.1

(1) $$d=t\sqrt{\frac{\left(n_{t}+n_{c}\right)}{\left(n_{t}n_{c}\right)}},$$

where *t *is the usual *t*-statistic given in eq.2

(2) $$t=\frac{{\bar{X}}_{t}-{\hat{X}}_{c}}{\sigma \sqrt{\frac{1}{n_{t}}+\frac{1}{n_{c}}}},$$

and *n*_*t *_and *n*_*c *_are individual sample sizes for treatment and control groups.

The estimator in eq.1 estimates the effect size based on the difference between the average gene expression values in the treatment and control groups (${\bar{X}}_{t}$ and ${\bar{X}}_{c}$) divided by the pooled standard deviation *σ *expressed in eq.3

(3) $$\sigma =\sqrt{\frac{n_{t}S_{1}^{2}+n_{c}S_{2}^{2}}{n_{t}+n_{c}-2}},$$

where *S*_1 _and *S*_2 _are standard deviations of treatment and control groups.

Hegdes and Olkin [16] have suggested correcting the effect size given in eq.1 for the sample size bias as shown below

(4) $$d^{\prime }=d-\frac{\left(3d\right)}{\left(4n-9\right)},$$

where *n *= *n*_*t *_+ *n*_*c*_.

The estimated variance *σ*^2^(*d'*) for the unbiased effect size is given in [16] as

(5) $$\sigma^{2}(d^{\prime })=\frac{n_{t}+n_{c}}{n_{t}n_{c}}+\frac{{d^{\prime }}^{2}}{2(n_{t}+n_{c})}.$$

For studies that measure the mean difference between treatment and control groups using a direct design approach we introduce a new standardized index given in eq.6

(6) $$d^{\prime \prime }=t_{paired}\sqrt{\frac{1}{n}},$$

where n is the sample size and *t*_*paired *_is the the expression for the paired *t*-statistic given in eq.7

(7) $$t_{paired}=\frac{\bar{X}}{\sqrt{\frac{\sigma_{paired}^{2}}{n}}},$$

where $\bar{X}$ is the mean difference between treatments and control for one sample data, $\sigma_{paired}^{2}$ is the sample variance and *t*_*paired *_is the Student *t *quantile with (*n *- 1) degrees of freedom.

Because *t*_*paired *_follows a *t *distribution with (*n *- 1) degrees of freedom, *d" *is distributed as $\sqrt{\frac{1}{n}}$ times a *t*-variate with (*n *- 1) degrees of freedom. Thus the mean and the variance of the standardized index *d" *can directly be obtained from the mean and the variance of the *t*-distribution as shown in eq.8

(8) $$\{\begin{array}{ll} E(d^{\prime \prime })=E\left(t_{paired}\sqrt{\frac{1}{n}}\right)=\mu =0, & for\;\nu >1\\ \sigma^{2}(d^{\prime \prime })=\sigma^{2}\left(t_{paired}\sqrt{\frac{1}{n}}\right)=\frac{1}{n}\frac{\nu }{\left(\nu -2\right)}, & for\;\nu >1 \end{array}$$

where *ν *designate the number of degrees of freedom (*ν *= *n *- 1). The null hypothesis H_0 _tested by *t*_*paired *_is thus that of no differences between treatments and control for one sample data (i.e H_0_:*μ *= 0). We note that for studies with direct design *n*_*t *_and *n*_*c *_denote the number of co-hybridized treatment and control samples for each one of the Cy5 and Cy3 channels with *n*_*t *_= *n*_*c *_= *n*, where *n *designate the total number of array replicates.

In our implementation the correct specification of the class labels depends on the type of data analyzed. Thus on the basis of class labels specified, our algorithm identifies the two types of data automatically. Experiments using two channel arrays with direct design correspond to the one-class label case while experiments with two groups of data correspond to the two class label case. Depending on the data type given, a *t*-statistic is calculated using either a two sample Welch *t*-statistic for the two class label case or a paired *t*-statistic for a single class label case. In both cases the *t*-statistics is calculated using the mt.teststat.num.denum() function from the R package multtest [21].

#### Hierarchical model

The hierarchical effect size model proposed by Choi et al. [15] is given as

(9) $$\{\begin{array}{ll} y_{j}=\theta_{j}+\epsilon_{j}, & \epsilon_{j}\sim N(0,s_{j}^{2})\\ \theta_{j}=\mu +\delta_{j}, & \delta_{j}\sim N(0,\tau^{2}) \end{array}$$

where *y*_*j *_is the observed effect size in study *j*, *θ*_*j *_is the mean gene expression in study *j*, *μ *is the average measure of differential expression for each gene across datasets, *τ*^2 ^is the estimated between study variability and *s*_*j *_is the estimated within-study variance.

Let ${d^{\prime }}_{j}$ denotes the observed unbiased effect size in study *j *for the two group data case and ${d^{\prime \prime }}_{j}$ denotes the observed standardized index in study *j *for the one group data case. In our implementation *y*_*j *_from eq.9, designate either ${d^{\prime }}_{j}$ (see eq.4) for the two group data case or ${d^{\prime \prime }}_{j}$ (see eq.6) for the one group data case. In the same way *s*_*j *_is calculated using either the expression given in eq.5 for the two group data case or the expression given in eq.8 for the one group data case. For the rest of this section, depending on data type to be integrated, *y*_*j *_and *s*_*j *_will designate either the observed effect size given eq.4 and its variance given in eq.5 or the observed standardized score given in eq.6 and its variance given in eq.8.

Following Choi et al. [15] if *τ*^2 ^= 0 in eq.9, then a fixed effect model (FEM) is used, otherwise a random effect model (REM) is used. To asses whether FEM or REM is most appropriate the hypothesis *H*_0 _: *τ*^2 ^= 0 is tested using the Cochran Q statistics given in eq.10

(10) $$Q=\sum w_{j}{\left(y_{j}-{\hat{\mu }}_{FEM}\right)}^{2},$$

where *w*_*j *_= $s_{j}^{-2}$ and ${\hat{\mu }}_{FEM}$ is the weighted least square estimator that ignores between-study variability as given in eq.11

(11) $${\hat{\mu }}_{FEM}=\frac{\sum w_{j}y_{j}}{\sum w_{j}}$$

Under the null hypothesis that *τ*^2 ^= 0, the statistics Q follows a $\chi_{\left(l-1\right)}^{2}$ distribution where *l *designates the total number of experiments.

If the null hypothesis of *τ*^2 ^= 0 is rejected, then the between study variability is estimated using eq.12

(12) $$\tau^{2}=max\left\{0,\frac{\left(Q-(l-1)\right)}{\sum w_{j}-(\frac{\sum w_{j}^{2}}{\sum w_{j}})}\right\}.$$

In the case of random effect model (REM) ${\hat{\mu }}_{FEM}$ in eq.11 is now expressed as

(13) $${\hat{\mu }}_{REM}=\frac{\sum w_{j}^{REM}y_{j}}{\sum w_{j}^{REM}},$$

where $w_{j}^{REM}={\left(s_{j}^{2}+\tau^{2}\right)}^{-1}$. The variance of ${\hat{\mu }}_{FEM}$ is given in eq.14

(14) $$var({\hat{\mu }}_{REM})=\frac{1}{\sum w_{j}^{REM}},$$

the z score statistic under REM can then be calculated as shown in eq.15

(15) $$z=\frac{{\hat{\mu }}_{REM}}{s},s=\sqrt{var({\hat{\mu }}_{REM})}.$$

In order to estimate the statistical significance of integrated results a permutation-based method developed by Tusher et al. [35] was used. In our model the permutation method used for estimating the false discovery rate (FDR) depends on the type of class labels provided. For the single class labels the permutation method is based on the paired *t*-statistic while for the two class label case the Welch *t*-statistic is used.

Within each dataset *j *= 1, 2,...*l *containing *k *= 1, 2,..., *p *genes, for each permutation *b *= 1, 2,..*B*, randomized data $y_{jk}^{randb}$ and $s_{jk}^{randb}$ were generated and overall mean ${\hat{\mu }}_{k}^{randb}$ and variance (see eq.15) were estimated to generate random $z_{k}^{randb}$ scores. The *z*_*k *_scores were then ordered (*z*_1 _≤ ... ≤ *z*_*p*_) together with random $z_{k}^{randb}$ scores $\left(z_{1}^{randb}\leq \ldots \leq z_{p}^{randb}\right)$ and FDR was estimated using the expression in eq.16

(16) $$FDR=\frac{(1/B)\sum_{b}\sum_{\left(k\right)}I(|z_{\left(k\right)}^{randb}|\geq z_{th})}{\sum_{\left(k\right)}I(|z_{\left(k\right)}|\geq z_{th})},$$

where *z*_*th *_is the threshold on the z score statistic [15] (see eq.15), and where I() equals 1 if the condition in parenthesis is true, and 0 otherwise.

## Authors' contributions

IB proposed the statistical model, designed the study, conducted data analysis and drafted the manuscript. AME, JEH and IDM initiated the study. AME, IDM, ZZ, and MK participated in revising the manuscript. LC and BP were responsible for data acquisition. All authors read an approved the final manuscript.

## Supplementary Material

Additional file 1 **GO over-represented categories**. Contains the file of all GO over-represented (p value ≤ 0.05) BP categories obtained with the MAID model (see also Table 1). Click here for file
